# Potassium silicate improves cellular sodium homeostasis in wheat (*Triticum aestivum* L.) cultivars differing in salt resistance

**DOI:** 10.1111/plb.70190

**Published:** 2026-02-11

**Authors:** M. T. Javed, S. H. Morgan, S. Lindberg

**Affiliations:** ^1^ Department of Botany, Faculty of Life Sciences Government College University Faisalabad Pakistan; ^2^ Department of Ecology, Environment and Plant Sciences Stockholm University Stockholm Sweden; ^3^ Plant Botany Department, Faculty of Agriculture Cairo University Giza Egypt

**Keywords:** Cytosolic Na^+^ uptake, fluorescence ratio microscopy, salt stress, silicate, sodium channels

## Abstract

- Salinity causes negative impacts on crops. We investigated the effect of potassium silicate (K_2_SiO_3_) on the cytosolic uptake of sodium, [Na^+^]_cyt_, in mesophyll protoplasts of wheat cultivars differing in salt sensitivity.
- Protoplasts were isolated enzymatically from 1‐week‐old wheat seedlings of cvs. S‐24 (salt tolerant) and Vinjett (salt sensitive). Protoplasts were loaded with the acetoxymethyl ester of SBFI, and subjected either to NaCl (0, 50, or 100 mM), followed by silicate (0 or 1 mM), or to silicate followed by NaCl. The sodium‐fluorescence changes were quantified by epi‐fluorescence microscopy.
- The cytosolic [Na^+^] uptake was higher in cv. Vinjett (salt‐sensitive), than in cv. S24 (salt‐resistant), which remained low even at high NaCl level. When silicate was added to protoplasts before NaCl, a significant reduction in [Na^+^]_cyt_ was observed for both cultivars compared with no silicate, but silicate addition after NaCl was less effective. Silicate treatment during the cultivation also reduced [Na^+^]_cyt_ uptake. Pharmacological results indicate that Na^+^ uptake in cv. Vinjett is mediated by both non‐selective, NSCCs, and K‐selective channels, but in cv. S‐24 mainly by NSCCs. Silicate might inhibit Na^+^ influx by these channels.
- Our novel findings reveal that silicate reduces the cytosolic Na^+^ uptake in wheat mesophyll cells and that the silicate impact depends on the cultivars' exploited Na^+^ uptake proteins. Further investigation is needed to clarify the role of silicate on different Na^+^ uptake channels.

## Graphical abstract

Potassium silicate can decrease the uptake of sodium (Na^+^) into wheat cells by affecting the Na^+^ uptake channels and increasing ion homeostasis.

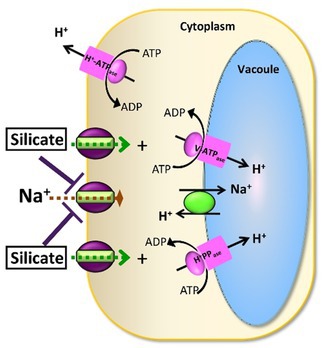

## INTRODUCTION

Soil salinity is a destructive threat to sustainable agriculture and crop productivity worldwide (Munns & Gilliham 2015; Zörb *et al*. 2019). Wheat (*Triticum aestivum* L.) is the staple food for half of the world's population and salt stress hampers its global production. Especially in arid and semi‐arid areas, salinity can be hazardous as it impairs water and nutrient uptake in plants (Munns & Tester 2008). If the stress is not severe, Na^+^ uptake can be mitigated by extra addition of nutritious elements like calcium and potassium (Morgan *et al*. 2013, 2014 and references therein), as well as silicon (Dhiman *et al*. 2021).

Silicon (Si) is the second most abundant mineral element in the soil after oxygen (Gong *et al*. 2006). This element is not considered ‘essential’ for plant growth but ‘quasi‐essential’ (Epstein & Bloom 2005), and its importance has been recognized for plants as it promotes structural stability, protects plants by improving resistance to abiotic and biotic stresses, and increases biomass production in some plants (Liang *et al*. 2015).

Silicon is taken up by plants in the form of silicic acid [Si(OH)_4_] if the solution pH is below 9 (Ma *et al*. 2006). The polymerization of silicic acid within the apoplast of plants during water loss by transpiration leads to formation of a colloidal silica gel (Exley 2015), which can alleviate salt‐stress by preventing sodium (Na) transport by the transpiration stream (Saqib *et al*. 2008).

Uptake of silicon into plant cells is mediated through protein transporters, that is, Lsi1, a low‐affinity transporter, which takes up Si into cortical cells and into the distal cells of the exo‐ and endodermis. In non‐accumulator plants like wheat, Lsi2 mediates xylem loading of Si by passive diffusion and Lsi6 is responsible for Si unloading from the xylem to the surrounding cells (Ma 2010). In many plant species, more than 90% of Si taken up by roots is translocated to shoots by the transpiration‐water flow and deposited as amorphous silica at transpiration sites, that is, leaves (Ma & Takahashi 2002).

Most of the beneficial effects of Si are attributed to its deposition in cell walls of the roots, leaves, and stems. Silicon mechanically supports the cell walls and membranes and mitigates structural damage caused by salinity (Li *et al*. 2024). Si is deposited below the thin cuticle layer and forms a cuticle–Si double layer (Ma & Yamaji 2015). Application of Si increases cell wall bound Na^+^ in the roots of salt‐tolerant and ‐sensitive wheat cultivars and reduces its root to shoot translocation (Saqib *et al*. 2008). It was reported that Si deposition in the wheat leaves limits transpiration, and hence salt accumulation (Bradbury & Ahmad 1990; Ahmad *et al*. 1992). Presence of Si might cause osmotic balance by ensuring cellular turgor and hydration under stress conditions (Shen *et al*. 2022; Irfan *et al*. 2023).

Uptake of Na^+^ into the plant cells is mediated by K^+^‐selective transporters as well as non‐selective cation channels, NSCCs, which could lead to a toxic cellular concentration of Na^+^ (Demidchik & Tester 2002). High‐affinity Na^+^ uptake was shown to be mediated by the HKT and HAK transporter families (Haro *et al*. 2010; Nieves‐Cordones *et al*. 2016; Lindberg & Premkumar 2024 and references therein), and HKTs can take up both K^+^ and Na^+^. The low‐affinity uptake of Na^+^ might be mediated by glutamate‐like receptors, cyclic‐nucleotide‐gated channels and NSCCs (Nieves‐Cordones *et al*. 2016). In rice, both HKTs and NSCCs were involved in Na^+^ uptake (Kader & Lindberg 2005; Kader *et al*. 2006). The *in planta* Na^+^ was considered to be more toxic than Cl^−^ as Na^+^ causes inhibition of enzyme activities, and the severity of such Na^+^ toxicity depends on the buildup of high cytoplasmic Na^+^ concentrations (Maathuis 2009). A decreased cytosolic Na^+^ concentration, either by Na^+^ efflux to the apoplast, or transport from cytosol into the vacuole, is suggested to be the main salt‐tolerance mechanisms in plants (Munns & Tester 2008), but also a plant's capability to maintain a high K^+^/Na^+^ concentration ratio in the cytosol is important (Morgan *et al*. 2013). Liang *et al*. (2006) reported that Si application might enhance the H^+^‐ATPase activity in the plasma membrane and tonoplast, which facilitates Na^+^ transport out of the cell's cytosol.

In saline soils, both sodium and chloride are present at high concentrations. Chlorine is an essential micronutrient for higher plants and is available in soils as chloride ion (Cl^−^). It has many functions in plants besides in photosynthesis, in nutrition, for instance as turgor‐ and osmose regulator, and as enzyme‐ and pH regulators (White & Broadly 2001). Experiments using *Vicia faba* showed that high concentration of Cl^−^ can be as toxic as high Na^+^ (Tavakkoli *et al*. 2010). Chloride can cause a reduction in growth and yield and might be underestimated as a toxic element under salinity. Plant roots can transport chloride across the plasma membrane in a passive way and both actively and passively across the tonoplast under saline condition (White & Broadly 2001). Under non‐saline conditions, Cl^−^ can be taken up in root cells by Cl^−^/2H^+^ symport, as well as by channels, although Cl^−^ – channels often mediate efflux of chloride ions (Barbier‐Brygoo *et al*. 2000; White & Broadly 2001). Elzenga & Van Volkenburgh (1997) reported a voltage‐dependent anion channel in mesophyll cells, activated by increase of cytosolic calcium. In wheat plants, the genes for the transporters TaNPF2.4/2.5 and TaCLC1, the latter likely a Cl^−^/2H^+^ channel, were identified (Buchner & Hawkesford 2014; Mao *et al*. 2022) and were induced by salt stress (Premkumar *et al*. 2022).

In previous studies, the role of silicon in alleviating salt stress in wheat at organ and tissue levels was investigated (Hanafy *et al*. 2008; Azeem *et al*. 2015). However, information is lacking concerning the role of silicate during the intracellular uptake of Na^+^ into wheat, which is important, as high Na^+^ concentration in the cytosol is toxic. Therefore, this investigation focuses on the cytosolic sodium uptake into protoplasts of two wheat cultivars, the salt tolerant cv. S‐24 (Ashraf  2010) and the moderate salt sensitive cv. Vinjett (Morgan *et al*. 2013) in the absence and presence of potassium silicate, K_2_SiO_3_. The cv. S‐24 is suggested to be an excluder and has been recommended for cultivation on saline soils. Our previous investigation (Premkumar *et al*. 2022) reported that silicate prevents the uptake of chloride into protoplasts of the same wheat cultivars by use of the chloride‐sensitive fluorescent dye MQAE.

Here, we investigated the impact of silicate presence during the cytosolic uptake of Na^+^ into the protoplasts and if silicate in the cultivation medium could affect the uptake of Na^+^. The influx of Na^+^ was measured by use of the acetoxy methyl ester of the Na^+^ − specific dye SBFI (SBFI‐AM) and by dual wavelength photometry. Pharmacological studies were executed to elucidate if silicate affects different Na^+^ channels/transporters in these cultivars. The outcome of this study could give valuable knowledge when cultivating crops on saline soil.

## MATERIALS AND METHODS

### Plant material and cultivation

Seeds of two wheat (*Triticum aestivum* L.) cultivars, cv. Vinjett (Svalöf‐Weibull, Sweden) and cv. S‐24 (Ayub Agricultural Research Institute, Faisalabad, Punjab, Pakistan) were treated with 10% chlorine solution for surface sterilization and then rinsed with distilled water 5–6 times. Thereafter, the seeds were soaked for 3 h in 5 mM CaSO_4_ solution and again rinsed 5–6 times with distilled water. The seeds were grown under dark conditions on a Mira cloth (LIC, Stockholm, Sweden) covering a metal net in a 1‐L beaker with a complete nutrient solution (2 mM KNO_3_,1 mM Ca(NO_3_)_2_, 1 mM MgSO_4_, 1 mM KH_2_PO_4_, 0.5 mM Na_2_HPO_4_, 2.5 μM H_3_BO_3_, 0.3 μM CuSO_4_, 0.5 μM ZnSO_4_, 2 μM MnSO_4_, 0.01 μM (NH_4_)_6_Mo_7_O_24_ and 200 μM Fe‐EDTA) (Shishova & Lindberg 2004). The beakers were covered with white polyethene and placed in a growth chamber equipped with 400 WHQI‐BT lamps (Osram, Munich, Germany). The growth chamber temperature was maintained at 21 ± 1 °C with 14 h photoperiod at an irradiance of 200 μmol m^−2^s^−1^ with relative humidity of about 60%.

The seedlings were grown with and without 1 mM K_2_SiO_3_ added to nutrient solution for separate experiments.

### Protoplast extraction

To measure cytosolic Na^+^ influx, protoplasts were used instead of cells, because esterases are present both in the cytosol and in the cell walls (Micheli 2001), where they can split the methyl ester dye into the sodium‐binding form and increase fluorescence also in the cell walls. The protoplasts were prepared from leaves of seven‐day‐old seedlings of both cultivars (Vinjett and S‐24). Younger leaves were sliced transversely into pieces smaller than 1 mm and about 1 g of the leaf material was incubated in 10 ml enzyme solution, containing 1% cellulase (Sigma Aldrich), 0.3% Macerozyme R‐10 (macerase Serva, Heidelberg, Germany), 0.5 M sorbitol 1 mM CaCl_2_, 0.2% bovine serum albumin (BSA, Sigma Aldrich), 0.05% polyvinyl pyrrolidone (PVP; Sigma Aldrich) and 20 mM MES (Sigma Aldrich) in a buffer at pH 5.5 (Lindberg & Strid 1997).

The incubation was carried out at 30 °C for 2.5–3 h in darkness and the suspension was carefully shaken to release the protoplasts and then filtered through a tea strainer. The filtrate was gently washed twice with 1 ml of the same solution as described above, but without enzymes (Medium 1), to release more protoplasts and thereafter filtered through a nylon net with 60 μm pores. The filtrate was centrifuged for 6 min at 42×*g* and the supernatant was removed with a suction pump. The pellet was suspended in 4 ml medium containing 0.5 M sucrose, 1 mM CaCl_2_ and 5 mM TRIS buffer at pH 7.0 (Medium 2). The suspension was then topped with a layer of 0.5 ml medium containing 0.4 M sucrose, 1 mM CaCl_2_, 0.1 M sorbitol and a 5 mM TRIS buffer at pH 7.0 (Medium 3) and a 0.5 ml layer of Medium 1. The protoplasts were collected from the intermediate green layer after centrifugation at 240×*g* for 5 min. The protoplast suspension was centrifuged for 6 min at 60×*g* and the pellet was used for dye loading.

### Dye loading

For measurement of cytosolic sodium concentration, [Na^+^]_cyt_, the protoplasts were loaded with the sodium‐binding acetoxy methyl ester of the benzofuran isophthalate dye, SBFI‐AM (Molecular Probes, Eugene, OR, USA), according to the method of Kader & Lindberg (2005). The protoplasts were incubated in the dye loading medium, which was composed of 0.5 M sorbitol, 0.1 mM CaCl_2_, 0.2% (w/v) polyvinyl pyrrolidone (PVP; Sigma), 5 mM TRIS and 5 mM MES at pH 5.5 (Medium A). The SBFI‐AM was dissolved in dimethyl sulphoxide (DMSO, Merck, Eurolab AB, Stockholm, Sweden; <0.1% water) to give a 5 mM stock solution. Two microlitre of the stock solution was mixed with 6.75 μl ethanol (Kemetyl, Stockholm, Sweden) and 1.25 μl pluronic F‐127 (Molecular Probes) and added to 1 ml of protoplast suspension to get a final concentration of 10 μM. Dye loading was carried out for 4 h at room temperature under darkness. The non‐specific esterases cleaved the acetoxymethyl (AM) groups of the dye inside the cytoplasm leaving it in a charged form which does not leak out of the cells.

After loading, the samples were centrifuged and pellets were resuspended into 1 ml of a solution similar to the loading medium, but with TRIS‐MES buffer at pH 7 (Medium B). The samples were kept at room temperature under darkness for 30 min before starting the measurements.

It was carefully checked that the protoplasts loaded with the SBFI dye and used for measurements showed fluorescence only from the cytosol, not from vacuoles. Protoplast's viability was investigated by use of Trypan Blue (Phillips 1973). Wheat protoplasts used for measurements had a viability of 91.8 ± 3%. At the end of all measurements after Na^+^ additions, protoplasts were re‐checked and considered only if they looked normal. In all experiments, silicon was added as K_2_SiO_3_.

### Fluorescence measurements

The fluorescence intensity of SBFI loaded protoplasts was measured by using an epi‐fluorescence microscope (Axiovert 10; Zeiss, Oberkochen, Germany), supplied with an electromagnetic filter‐exchanger (Zeiss), Xenon lamp (Zeiss XBO 75), microprocessor (MSP 21, Zeiss), photometer (Zeiss 01) as well as a personal computer after excitation at 340/380 nm, which represent the upper and lower points of the SBFI dye excitation spectrum. Emission wavelengths during the measurements were 530–550 nm. A Planneofluar 40/0.75 objective (Zeiss) was used for all measurements and phase contrast. The signals and noise adjustment was made automatically. The effect of different dye concentrations can be eliminated by means of dual wavelength microscopy.

To attach the protoplasts to a glass surface, the microslides were covered with 0.2% poly‐L‐lysine (MW 150,000–300,000, Sigma).

#### *In situ* calibration

For standard determination of cytosolic Na^+^, an *in situ* calibration of SBFI‐AM fluorescence ratio 340/380 nm was performed with different protoplasts incubated in TRIS‐MES buffer (pH 7) including 0, 25, 50, 75, and 100 mM NaCl as described by Kader & Lindberg (2005). The *in situ* calibration measurement of fluorescence intensity at 340/380 nm at different NaCl concentrations shows a linear relationship between 10 and 100 mM Na (Fig. S1).

#### Fluorescence measurements after silicate and NaCl additions

The fluorescence intensity ratio of the cytosol at 340/380 nm was determined in single protoplasts before, and after, addition of 1 mM K_2_SiO_3_ and 50 or 100 mM NaCl to the protoplast suspension. Fifty measurements were taken by a photometer every 250 ms; the traces show the mean of these measurements from a typical experiment. The traces' variance was less than 5%. By means of regression analysis of the *in situ* calibration curve, fluorescence intensity ratios were converted to the cytosolic Na^+^‐concentration. Measurements were carried out with protoplasts of similar size that exhibited fluorescence from the cytosol only. After addition of K_2_SiO_3_, the low concentration of K^+^ (2 mM) compared with Na^+^ (50 or 100 mM) does not affect the SBFI fluorescence, as SBFI is 18‐fold more selective for Na^+^ than K^+^ (Minta & Tsien 1989).

### Statistical analysis

Each plot is a copy of printer plots and shows representative traces of a specific experiment repeated more than 10 times with protoplasts from independent cultivations. Each value is the average of around 50 fluorescent‐ratio determinations. The Figs. 3 and 4 and the table also show data from experiments repeated up to 10 times with protoplasts from independent cultivations. Statistical analysis of data was performed with analysis of variance (ANOVA) by using a statistical program Co‐Stat version 6.2, Cohorts Software, 2003, Monterey, CA, USA. Tukey's test (HSD‐test) was used for detection of differences among the treatment means (*P* ≤ 0.05).

## RESULTS

Mesophyll protoplasts isolated from the leaves and loaded with the fluorescence dye SBFI‐AM were used to measure the changes in cytosolic Na^+^ concentration upon addition of NaCl in the absence or presence of potassium silicate (K_2_SiO_3_).

### Silicate addition before NaCl

**Fig. 1:**
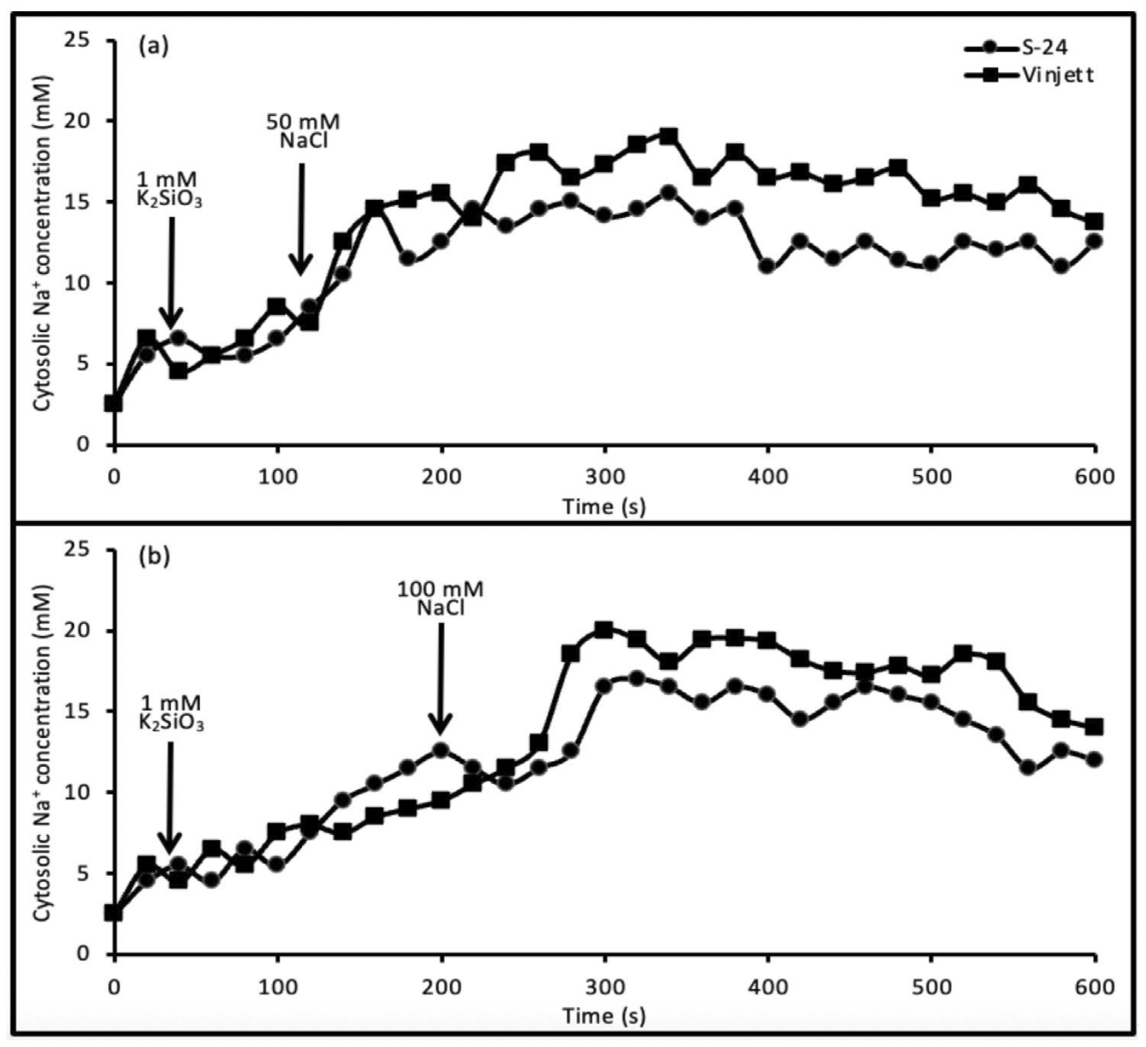
Changes of cytosolic Na^+^ concentration in leaf mesophyll protoplasts of cv. Vinjett and cv. S‐24 with time. Addition of 1 mM K_2_SiO_3_ to protoplasts was followed by 50 mM NaCl addition (a) or by 100 mM NaCl addition (b). Different additions are indicated by arrows. Changes in fluorescence intensity ratio 340/380 nm, measured every 2.5 s, were averaged and converted to cytosolic Na^+^ concentration by using an *in situ* calibration curve. Typical traces (variance was less than 5%) representative of 10 experiments with protoplasts from independent cultivations.

When 1 mM silicate was added to the protoplasts before addition of 50 or 100 mM NaCl, the cytosolic Na^+^ concentrations, [Na^+^]_cyt_, increased in both the cultivars but at differential levels (Fig. 1a,b). After the optimal increase, the traces levelled off for both cultivars.

After 1 mM K_2_SiO_3_ and 50 mM NaCl addition, the [Na^+^]_cyt_ was 8.69 mM in cv. S‐24, which was 30% lower than in cv. Vinjett, at the end of the measurement when the curve was stabilized (Table 1). The [Na^+^]_cyt_ was 52% and 50% lower for the cvs. Vinjett and S‐24, respectively, as compared with protoplasts that were not treated with silicate.

**Table 1:** Changes in cytosolic Na^+^ concentration, [Na^+^]_cyt_, in leaf protoplasts of cv. Vinjett and cv. S‐24 upon addition of 1 mM K_2_SiO_3_ followed by 50 or 100 mM NaCl addition. Data are also shown when 50 or 100 mM NaCl addition was added before 1 mM K_2_SiO_3_ to the protoplasts of both the cultivars. Letters (a–f) indicate significant difference (*P* ≤ 0.05) in [Na^+^]_cyt_ after different salt additions with and without K_2_SiO_3_. Letters (y,z) represent significant difference (*P* ≤ 0.05) in [Na^+^]_cyt_ for cv. Vinjett and cv. S‐24 subjected to the same treatment. Mean ± SE of 10 experiments repeated with protoplasts from independent cultivations.

treatments		changes in cytosolic Na^+^ concentration (mM)	
1st addition to protoplast	2nd addition to protoplast	cv. Vinjett	cv. S‐24
0 mM K_2_SiO_3_	50 mM NaCl	25.41 ± 0.306^cy^	17.31 ± 0.212^cz^
	100 mM NaCl	35.68 ± 0.385^ay^	26.49 ± 0.333^az^
1 mM K_2_SiO_3_	50 mM NaCl	12.32 ± 0.262^ey^	8.69 ± 0.217^fz^
	100 mM NaCl	16.10 ± 0.464^dy^	11.5 ± 0.288^ez^
50 mM NaCl	0 mM K_2_SiO_3_	25.41 ± 0.306^cy^	17.31 ± 0.212^cz^
	1 mM K_2_SiO_3_	24.33 ± 0.366^cy^	14.89 ± 0.295^dz^
100 mM NaCl	0 mM K_2_SiO_3_	35.68 ± 0.385^ay^	26.49 ± 0.333^az^
	1 mM K_2_SiO_3_	32.99 ± 0.202^by^	23.39 ± 0.230^bz^

When 100 mM NaCl was added after 1 mM silicate, the [Na^+^]_cyt_ in cv. Vinjett and cv. S‐24 was 16.1 and 11.5 mM, respectively. Thus, the [Na^+^]_cyt_ of both the cultivars, after NaCl addition, was significantly lower than the concentration in protoplasts without silicate pretreatment (Table 1).

### NaCl addition before silicate

**Fig. 2:**
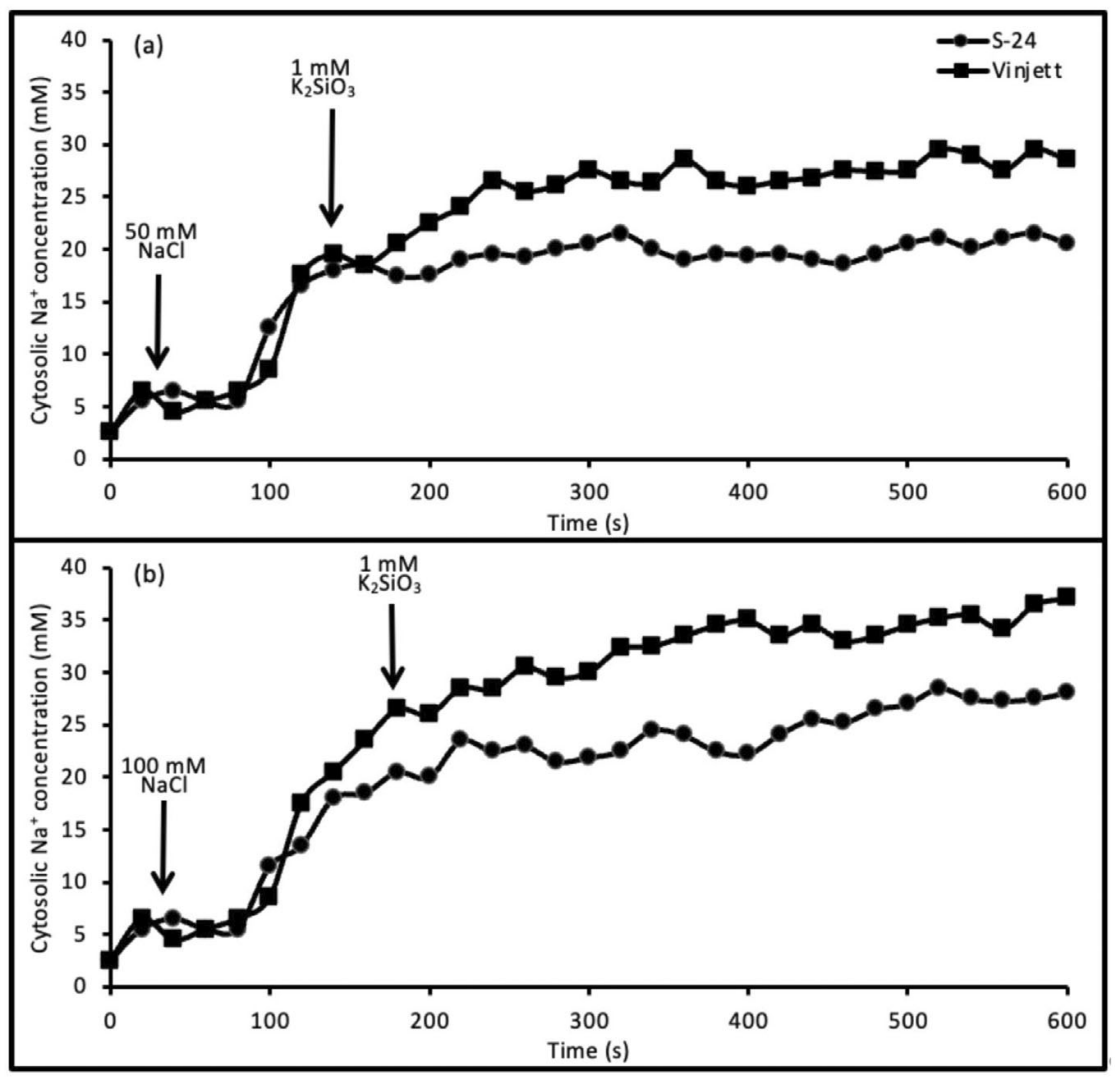
Changes of cytosolic Na^+^ concentration in mesophyll protoplasts of cv. Vinjett and cv. S‐24 with time. NaCl, 50 mM (a) or 100 mM (b) was added before 1 mM K_2_SiO_3_. Different additions are indicated by arrows. Changes in fluorescence intensity ratio 340/380 nm, measured every 2.5 s, were averaged and converted to cytosolic Na^+^ concentration by using an *in situ* calibration curve. Typical traces (variance was less than 5%) representative of 10 experiments with protoplasts from independent cultivations.

When NaCl instead was added before 1 mM silicate to protoplasts, silicate was less effective in reducing the [Na^+^]_cyt_ of both the cultivars (Fig. 2a,b, Table 1). When protoplasts were treated with 50 or 100 mM NaCl prior to 1 mM silicate addition, the cytosolic Na^+^ concentration exhibited a steady increase with time both in cv. Vinjett and cv. S‐24 (Fig. 2a,b). After addition of 50 mM NaCl followed by silicate, the cytosolic Na^+^ concentration was approximately 14.9 mM in cv. S‐24, which was 39% lower than in cv. Vinjett (Table 1). Then a significant reduction of [Na^+^]_cyt_ was noted only for cv. S‐24, which was 14% lower than at 50 mM NaCl alone. However, after 100 mM NaCl addition small significant reductions of [Na^+^]_cyt_ by silicate were obtained in both the cultivars.

### Cultivation of seedlings with or without silicate before the uptake experiments

**Fig. 3:**
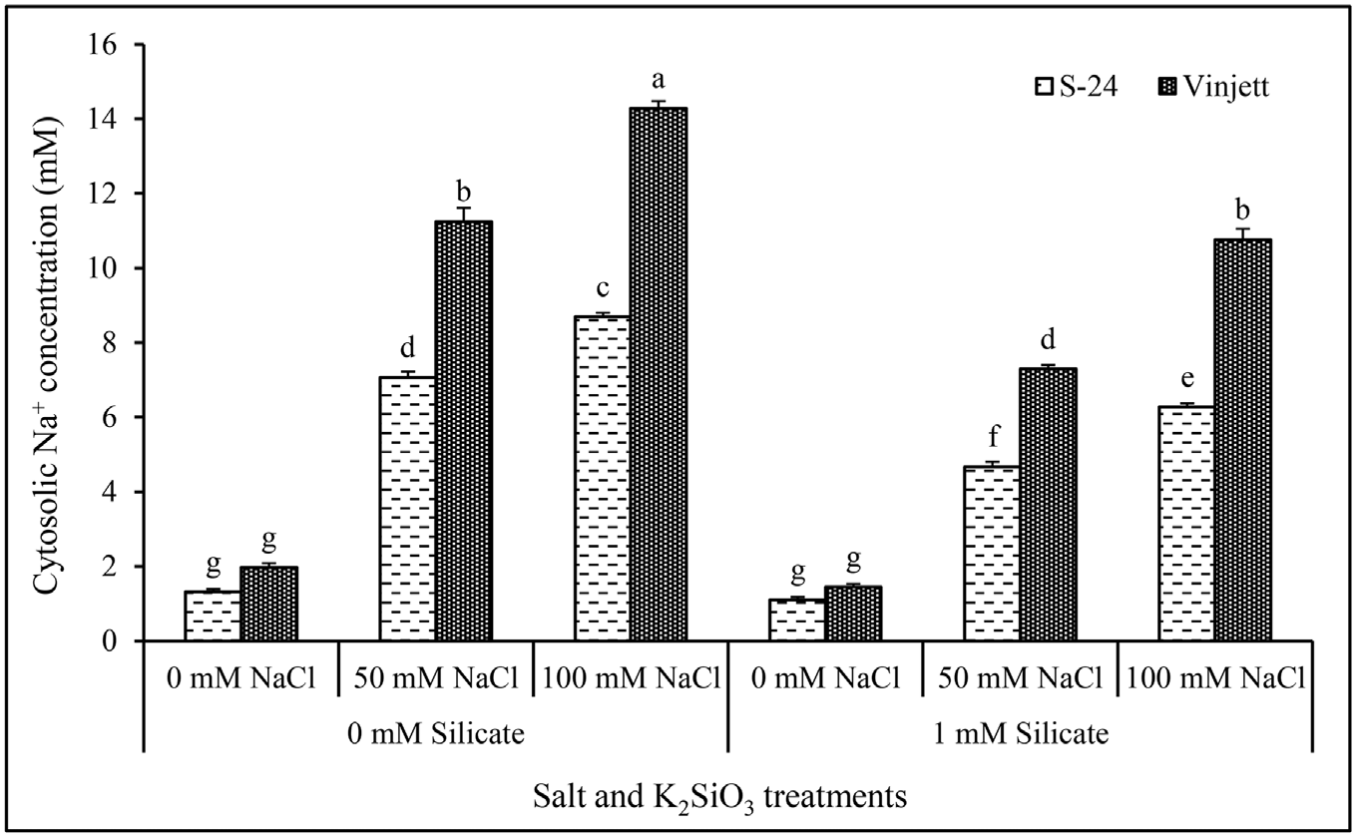
Changes in cytosolic Na^+^ concentrations in mesophyll protoplasts of cv. Vinjett and cv. S‐24 upon addition to protoplasts of 0, 50 and 100 mM NaCl. Before protoplasts isolation, both cultivars were grown in nutrient solutions supplemented with or without 1 mM K_2_SiO_3_. Letters (a–g) indicate significant difference (*P* ≤ 0.05) in [Na^+^]_cyt_ after different salt additions to protoplasts obtained from plants grown with or without K_2_SiO_3_. Data represent the mean ± SE of 10 experiments with protoplasts from independent cultivations.

As silicate addition prior to NaCl stress was more effective to minimize the salt stress than when NaCl was added first, we also performed experiments with seedlings cultivated in a nutrient solution with or without 1 mM K_2_SiO_3_. The results demonstrated that the uptake of Na by protoplasts of cv. Vinjett exceeded that of cv. S‐24, both at 50 and 100 mM NaCl addition to protoplasts, irrespective of cultivation with or without silicate, although the uptake was lower after cultivation with silicate (Fig. 3). At 50 mM NaCl addition, the [Na^+^]_cyt_ of cv. S‐24 was only 7.06 mM, which was 37% lower than in cv. Vinjett.

After cultivation with silicate and 100 mM NaCl addition, the [Na^+^]_cyt_ of cv. Vinjett and cv. S‐24 protoplasts was 10.75 and 6.27 mM, respectively, which was 23% and 28% lower as compared with those without silicate pretreatment.

In cv. Vinjett protoplasts from seedlings cultivated *without* silicate, addition of 50 or 100 mM NaCl to protoplasts exhibited about 6–7‐fold increase in [Na^+^]_cyt_, as compared with control (without NaCl), and approximately 4–5 fold after cultivation *with* silicate (Fig. 3). Similar results were obtained for cv. S‐24, although at lower levels of [Na^+^]_cyt_.

### Pharmacological experiments

A pharmacological approach was used to investigate which ion transporters could mediate the influx of Na^+^ into the two cultivars. Inhibitors for K‐selective transporter proteins, such as Ba^2+^, barium, and TEA, tetraethylammonium, and an inhibitor to NSCCs, non‐selective cation channels, such as La^3+^, lanthanum, were added to the protoplasts of both cultivars 15 min before addition of 50 or 100 mM NaCl.

**Fig. 4:**
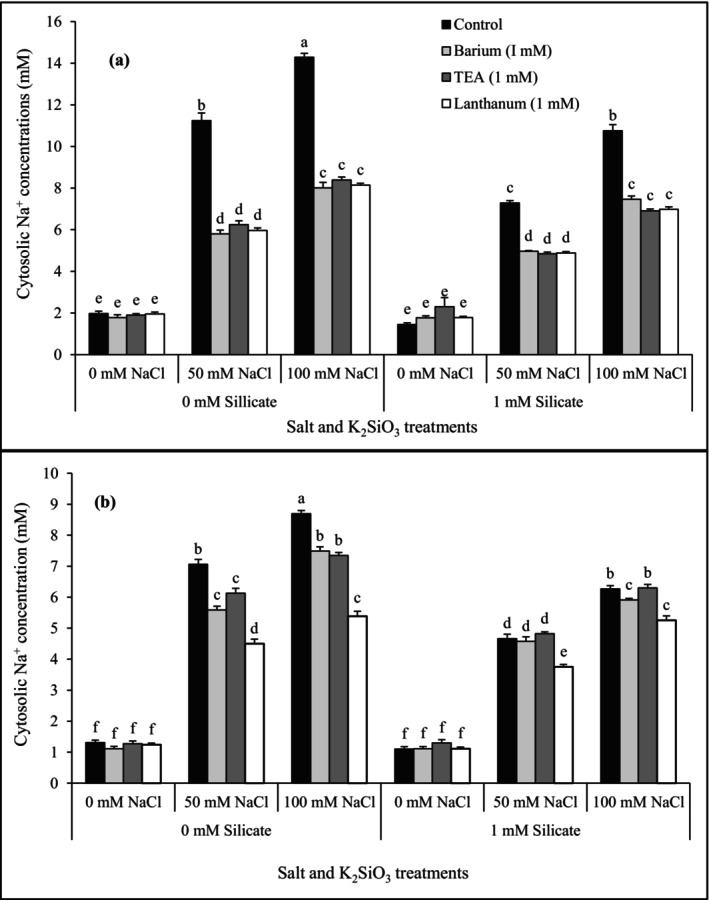
Effects by different transporter inhibitors on the cytosolic Na^+^ concentration in mesophyll protoplasts of cv. Vinjett (a) and cv. S‐24 (b) upon addition of 0, 50, and 100 mM NaCl. Before the salt additions, protoplasts were pretreated 15 min with 1 mM Ba^2+^, 1 mM TEA, or 1 mM La^+^. Seedlings were cultivated in full nutrient solution with or without 1 mM K_2_SiO_3_ before preparation of protoplasts. Fluorescence intensity ratios taken at 340/380 nm after the NaCl additions were converted to cytosolic Na^+^ concentration by using the *in situ* calibration curve. Letters (a–f) indicate significant difference (*P* ≤ 0.05) in [Na^+^]_cyt_ between different inhibitor treatments and the control (no inhibitor). Data represent the means ± SE of 10 experiments with protoplasts from independent cultivations.

With no NaCl addition, the cytosolic concentration of Na^+^ in the protoplasts of both cvs. pretreated with Ba^2+^, TEA, and La^3+^ was approximately the same, 2 mM, in cv. Vinjett and about 1.2 mM in cv S‐24, with or without 1 mM silicate during cultivation (Fig. 4a,b).

Pretreatment of cv. Vinjett protoplasts from seedlings cultivated *without* silicate with 1 mM of Ba^2+^, TEA or La^3+^, before addition of 50 or 100 mM NaCl, significantly decreased the [Na^+^]_cyt_, as compared with non‐treated controls (without inhibitors) (Fig. 4a). At 100 mM NaCl addition, the reduction of [Na^+^]_cyt_ was 44%, 41%, and 43% after pretreatment with the respective inhibitors. Moreover, when silicate was present during cultivation, the inhibitors' mediated reduction of [Na^+^]_cyt_ in cv. Vinjett followed the same trend when NaCl was added to protoplasts (Fig. 4b).

In cv. S‐24, addition of 50 or 100 mM NaCl to protoplasts caused a lower increase of [Na^+^]_cyt_ than in cv. Vinjett (Fig. 4a,b). The inhibitors Ba^2+^, TEA, and La^3+^ differently affected the [Na^+^]_cyt_ of protoplasts after cultivation with or without silicate (Fig. 4b). When seedling were cultivated without silicate, treatment of protoplasts with 1 mM Ba^2+^ or TEA, caused a significant reduction of [Na^+^]_cyt_, while reduction by La^3+^ was more pronounced when all three inhibitor effects were compared with untreated controls (without inhibitors). Lanthanum reduced the [Na^+^]_cyt_ up to 38% after both 50 mM and 100 mM NaCl addition, respectively. Moreover, when the cv. S‐24 was cultivated in the presence of silicate, the uptake of Na^+^ in the protoplasts at 50 mM addition, was inhibited only by 1 mM La^3+^ and resulted in 3.75 mM [Na^+^]_cyt_ at 50 mM NaCl and 5.25 mM at 100 mM NaCl addition, as compared with their respective untreated controls. Beside La^3+^ inhibition, also a small, but significant reduction with Ba^2+^ was obtained at addition of 100 mM NaCl to cv. S‐24 (Fig. 4b).

## DISCUSSION

The findings that the salt‐resistant wheat, cv. S‐24, takes up less Na^+^ in the cytosol than the moderate sensitive wheat cultivar, cv. Vinjett, upon short‐term NaCl treatment (Table 1), are in agreement with earlier findings of rice and wheat (Kader & Lindberg 2005; Morgan *et al*. 2013). Similarly, Carden *et al*. (2003), by use of triple‐barreled microelectrodes, found a 10‐fold lower Na^+^ activity in a tolerant barley variety than in a sensitive one. Sodium efflux from the cytosol into the apoplast and/or sequestration to intracellular compartments (*i.e*. vacuoles) is an important salt‐tolerance mechanism in plants. The low Na^+^ concentration in the salt tolerant cv. S‐24 limits Na^+^ toxicity and enables it to grow in saline environments (Ali *et al*. 2008; Ashraf & Ashraf 2016). The later authors concluded that cv. S‐24 showed a better physiological performance, for example, longer green leaves under salt stress at different stages compared with the salt sensitive cultivar.

### Addition order of silicate and NaCl is important

Many studies reported that Si presence causes reduction in Na uptake in salt‐stressed plants at organ or tissue levels (Gong *et al*. 2006; Ashraf *et al*. 2010; Liang *et al*. 2015). Here, we report that silicate application to wheat protoplasts decreases the cytosolic Na^+^ influx in both cultivars in a similar way, and that silicate addition before NaCl addition is more effective than the opposite order. This could depend on silicate inhibition of Na^+^‐binding to the Na^+^ uptake sites on the plasma membrane, which in turn reduces Na^+^ transport to the inner side of the membrane, as reported for silicate inhibition of Cd uptake (Greger *et al*. 2016). Furthermore, silicate might also change the conformation of the plasma membrane, and thus, change its ion transport mechanisms.

After pretreatment with silicate, the cytosolic influx of Cl^−^ in the same wheat cultivars (cvs. Vinjett and S‐24) was diminished in a similar way as for Na^+^ (Premkumar *et al*. 2022). It is therefore likely that silicate affects both the plasma membrane and the ion transporters.

The more noticeable reduction obtained when silicate was added before NaCl might also be due to a silicate activation of the tonoplast H^+^ATPase and H^+^PPase (Liang *et al*. 2005; Dhiman *et al*. 2021), as silicate easily can be transported through the plasma membrane and into the cytosol as shown for barley and aloe (Liang *et al*. 2006; Xu *et al*. 2015).

Thus, the results confirm that silicate addition before salt treatment should be of importance for preventing cellular uptake of Na^+^.

### Silicate treatment during cultivation diminishes cytosolic Na^+^ influx in wheat

The more distinctive inhibition of the Na^+^ influx into protoplasts after cultivation in the presence of silicate, than without silicate, might depend on salt stress‐induced changes of the plasma membrane lipid composition and subsequent changes of ion transport (Yahya *et al*. 1995). Another reason is that silicate application could stimulate the synthesis of polyamines that might limit intracellular accumulation of Na^+^ as reported for root protoplasts of salt‐stressed barley (Zhao *et al*. 2007; Yin *et al*. 2016).

### Silicate effects on Na^+^ influx may depend on the involved transport proteins

By use of inhibitors during influx experiments, it was possible to suggest which transporters mainly mediated the cytosolic Na^+^ influx. As the inhibition of Na^+^ influx followed the same pattern in cv. Vinjett by all of the used inhibitors, both K^+^‐selective transporters and NSCCs likely were involved in mediating Na^+^ influx both with and without silicate presence during the cultivation (Fig. 4a,b). This could explain the high cytosolic influx of Na^+^ into cv. Vinjett.

In cv. S‐24 cultivated without silicate, pretreatment with either 1 mM Ba^2+^ or 1 mM TEA, as well as with La^3+^, the uptake of Na^+^ was significantly reduced upon salt addition, but more prominently by La^3+^. Therefore, K^+^‐selective transporters seem to be partly involved in cytosolic Na^+^ uptake also in cv. S‐24, although the main uptake occurs via NSCCs (Fig. 4b).

However, after addition of 100 mM Na^+^, the somewhat higher silicate effect on cv. S‐24 in comparison to cv. Vinjett might depend on the fact that silicate could more easily block the NSCCs channels than the K^+^‐selective transporters also involved in sodium uptake in cv. Vinjett.

On the other hand, the inhibitors, added at 1 mM, are not quite specific for these transport proteins. For instance, Ba^2+^ is considered to block K^+^‐selective channels but might also block NSCCs (Demidchik & Tester 2002). Tetra ethyl ammonium (TEA) is used as a blocker for K^+^‐selective channels but can also affect Na^+^ uptake. In rice, TEA inhibits Na^+^ uptake in a non‐competitive way, which can be interpreted as a TEA binding to the channel (Kader & Lindberg 2005).

Our results are in line with the studies using sensitive and resistant rice cultivars, which suggested that both types of transporters were mediating Na^+^ uptake in the sensitive cv. BRRI Dhan29, but mainly the NSCCs were involved in the uptake of Na^+^ into the resistant cv. Pokkali (Kader & Lindberg 2005).

Inward rectifying potassium transporters can mediate Na^+^ transport under high salt addition, although these transporters are highly selective for K^+^ (Amtmann & Sanders 1999). It was found that K^+^‐selective transporters might contribute to Na^+^ influx in the moderate salt sensitive cv. Vinjett, even in the presence of silicate. Other reports have stated that the cytosolic Na^+^ uptake across the plasma membrane in many plants can be mediated by high‐affinity potassium transporters of the HKT family (Horie *et al*. 2001; Golldack *et al*. 2002; Mian *et al*. 2011).

It is interesting to note that when cv. S‐24 plants were cultivated in the presence of silicate, the uptake of the Na^+^ into the protoplasts was only inhibited when pretreated with 1 mM La^3+^, which suggests that the cytosolic Na^+^ uptake in this cultivar is mainly mediated by NSCCs. These channels have previously been reported to be the major pathway for cytosolic Na^+^ influx in many plant species under salinity (Demidchik & Tester 2002; Essah *et al*. 2003).

## CONCLUSIONS

Our novel results show that cytosolic Na^+^ uptake is much lower in the salt‐resistant wheat cv. S‐24 than in the moderately salt sensitive cv. Vinjett. It might depend on that both K^+^‐selective transporters and NSCCs are involved in the Na^+^ uptake in that cultivar, while mainly NSCCs are responsible for Na^+^ influx in cv. S‐24.

Silicate addition to the protoplasts prior to salt treatment reduces [Na^+^]_cyt_ predominantly in the salt sensitive cultivar, and in a more effective way than if silicate is added after salt addition. By silicate presence in the cultivation medium, both cultivars can maintain a low [Na^+^]_cyt_ in the leaves, probably by a change in the transporter's function or by salinity induced changes in the plasma membrane.

Further experimental work is needed to investigate the role of silicate in cellular sodium uptake in these cultivars. It is likely that the outcomes will contribute to new strategies to grow wheat in salt‐affected areas.

## AUTHOR CONTRIBUTIONS

MTJ and SL wrote the paper, SHM and MTJ did calculations and figures. All authors read and corrected it. SL and MTJ planned the study.

## Supporting information


**Fig. S1.** *In situ* calibration curve of SBFI‐AM dye showing fluorescence intensity ratio at 340/380 nm when subjected to 0, 25, 50, 75, and 100 mM NaCl concentrations.
