# Supplementary material for: Potassium silicate improves cellular sodium homeostasis in wheat (Triticum aestivum L.) cultivars differing in salt resistance
Source: Plant Biol (Stuttg). 2026 Feb 11;28(4):1043–51. doi: 10.1111/plb.70190 (PMC13175953; doi:10.1111/plb.70190)

**Supplementary Figure**

**Fig. S1.** *In situ* calibration curve of SBFI-AM dye showing fluorescence intensity ratio at 340/380 nm when subjected to 0, 25, 50, 75, and 100 mM NaCl concentrations.


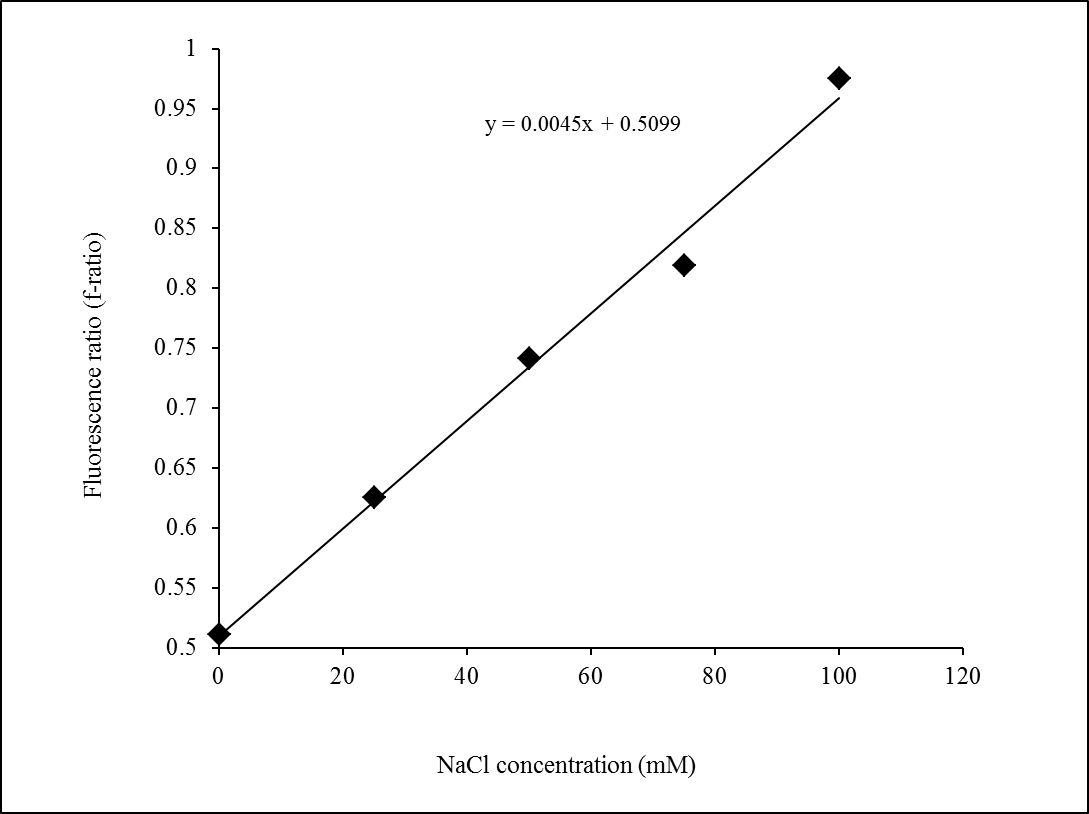

Supplement: Supplementary file 1 — Fig. S1. In situ calibration curve of SBFI‐AM dye showing fluorescence intensity ratio at 340/380 nm when subjected to 0, 25, 50, 75, and 100 mM NaCl concentrations. [file PLB-28-1043-s001.docx]
